# Supplementary material for: Unidirectional response to bidirectional selection on body size II. Quantitative genetics
Source: Ecol Evol. 2020 Oct 1;10(20):11453–66. doi: 10.1002/ece3.6783 (PMC7593195; doi:10.1002/ece3.6783)
Supplement: Supplementary file 2 — Appendix S1‐4 [file ECE3-10-11453-s002.pdf]

Appendix 1

Model convergence

Autocorrelation and effective size

	Lag 1	Lag 5	Lag 10	Lag 50	Lag 100	Lag 500	Eff. Size
V[G](Sdl)	0.99	0.96	0.93	0.72	0.52	-0.08	1312.64
V[G](Mat)	0.99	0.97	0.94	0.79	0.64	0.10	466.69
C[G](Sdl, Mat)	0.99	0.97	0.94	0.75	0.59	0.02	679.69
V[E](Sdl)	0.95	0.85	0.79	0.55	0.40	-0.06	1461.35
C[E](Sdl, Mat)	0.96	0.88	0.82	0.57	0.42	-0.02	1526.90

Autocorrelation for the random effect parameters was assessed with the autocorr.diag() function from package coda. The effective size (sample size adjusted for autocorrelation) was evaluated with the function effectiveSize() from the same package.

.

Stationarity

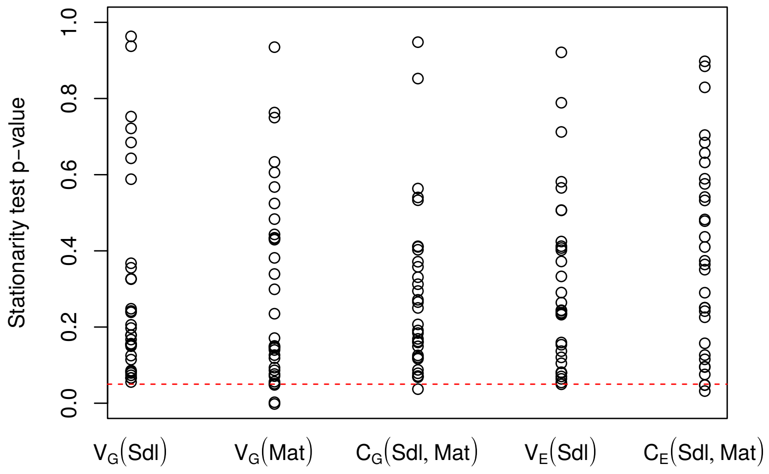

The vast majority of MCMC chains passed the Heidelberg stationarity test implemented in the heidel.diag() function from the coda package (null hypothesis H_0: the chain is stationary at least over its last half, the dashed line illustrates the 5% threshold).

Appendix 2

Inbreeding

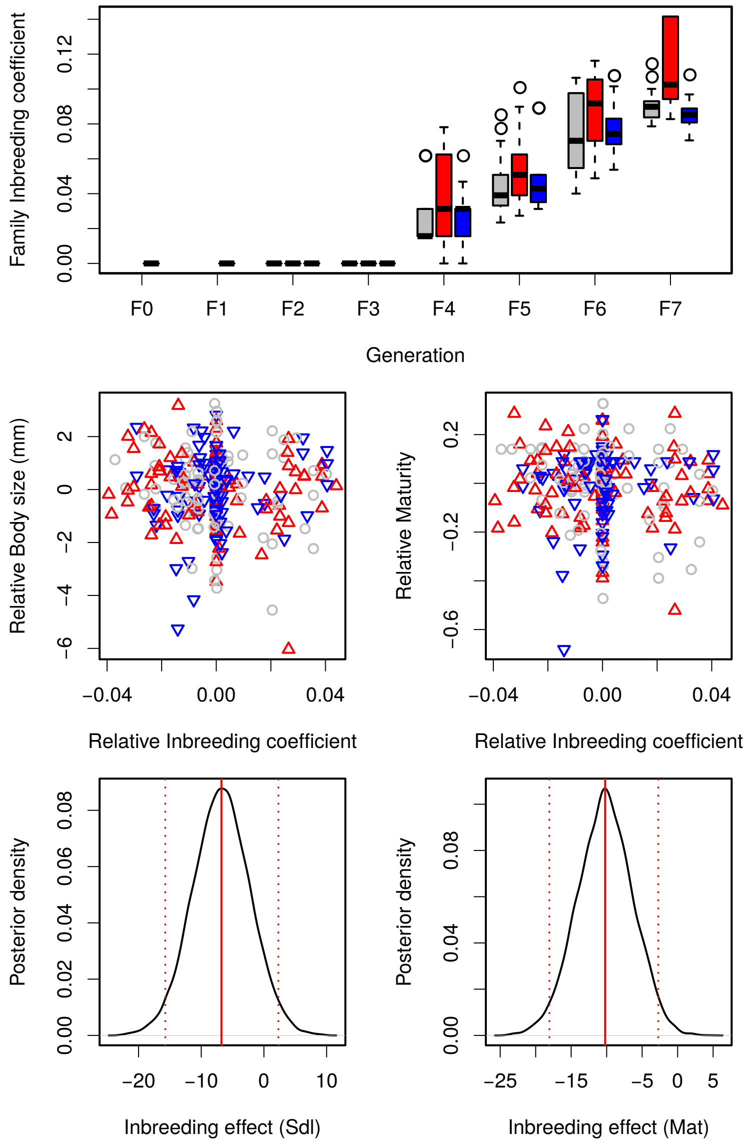

Top: Distribution of the inbreeding coefficients (calculated from the full pedigree) across families in the course of the experiment (assuming no inbreeding in F_0). Middle: relationship between the inbreeding coefficient of families (normalized by the average of the line each generation) to phenotypic traits (centered on the line and generation mean). None of these regressions were statistically significant. Bottom: posterior distribution of the inbreeding effects on Sdl and maturity (vertical lines indicate 2.5%, 50%, and 97.5% quantiles).

Appendix 3

Model fitting on partial datasets

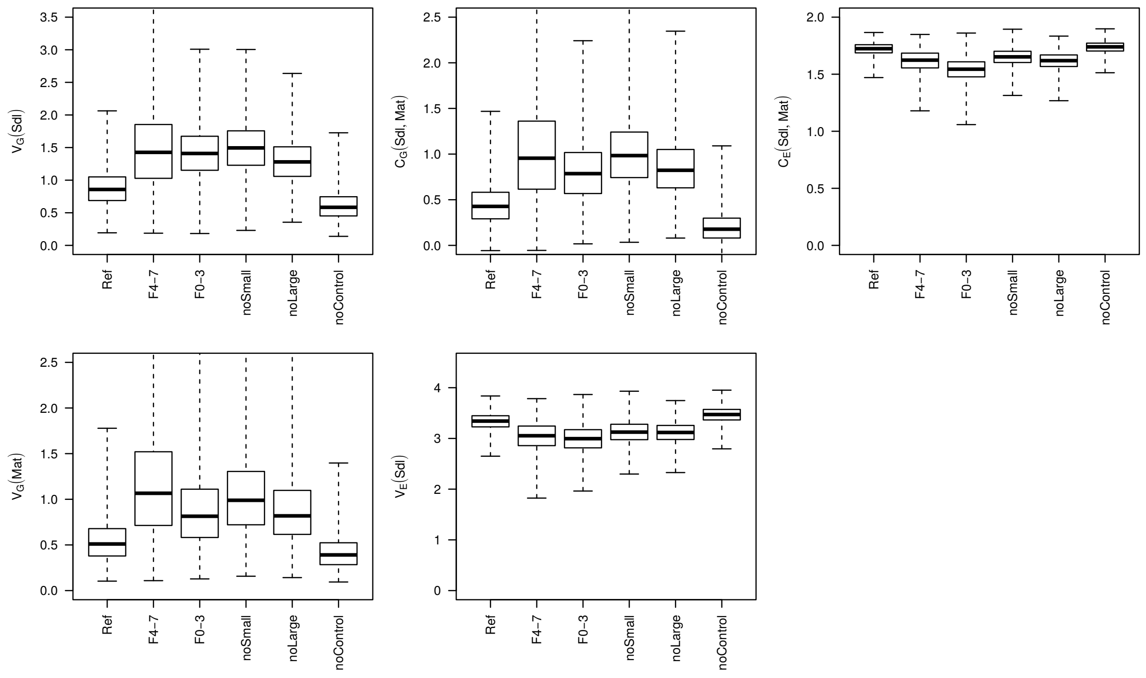

The animal model estimates variance components in the starting population (F_0) accounting for drift and selection in subsequent generations. As a consequence, if the assumptions of the infinitesimal model hold, fitting the model on partial datasets should not affect the estimates (while the posterior distribution is expected to be wider due to the decrease in information). We split the dataset according to (i) generations (fitting the model on generations F_0 to F_3, and from F_4 to F_7), and (ii) to the selected line (Large, Small, and Control lines), fitting the model excluding sequentially each line. In the figure, "Ref" stands for the posterior when including all the data, and boxplots represent the full range of the posterior distributions and their quartiles. The estimates for genetic variances and covariances increased for most sub-datasets, and residual variances and covariances decrease accordingly. The most straightforward explanation is that the parameters estimated from the full dataset result from a compromise between early/late generations and selection lines, and that the goodness of fit of the model increased when fitted on partial data. Note that most posterior distributions largely overlap (no posterior distributions differ significantly from the reference), suggesting that the estimated parameters remain meaningful.

Appendix 4

Parent-offspring regression for body size

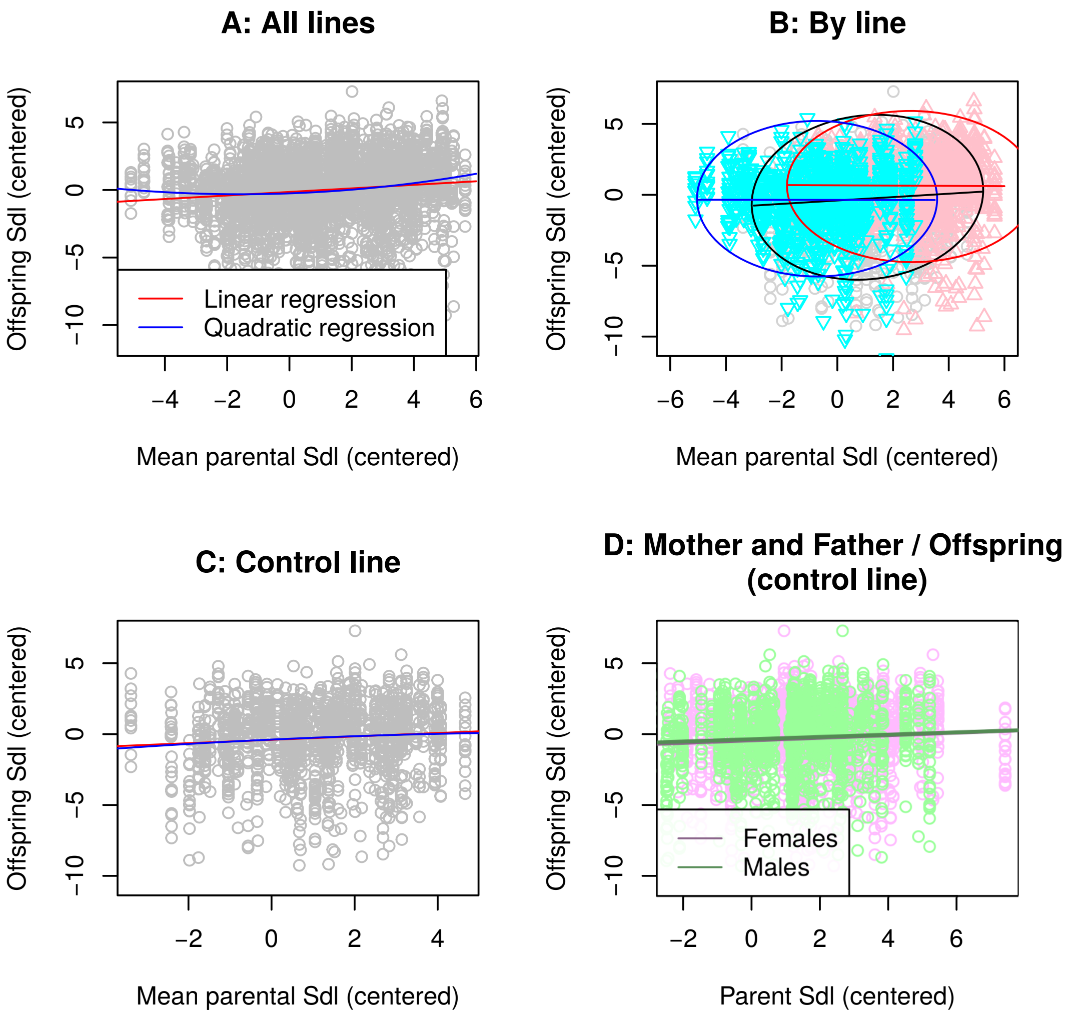

The mid-parent-offspring regression coefficient estimates trait heritability. In addition, the shape of the parent-offspring relationship is indicative of potential deviations from the infinitesimal model assumptions. Non-linear parent-offspring relationships may indicate dominance, epistasis, or genetic asymmetries.

A. Taking all selected lines into account, normalizing by generation phenotypic averages to cancel out generation effects, the parent-offspring relationship appeared to be slightly non-linear (significant quadratic component: $y = c + h^2x + k_2x$, with $h^2 = 0.083 \pm s.e. 0.021$ being an estimate of heritability ($\Pr(h^2 = 0) = 6.57 \cdot 10^{-5}$), the quadratic term being also significant ($\Pr(k_2 = 0) = 5.81 \cdot 10^{-5}$).

B. However, considering each line separately, the pattern rather reflected different linear relationships in all three lines. The Large line response to selection shifted the offspring phenotype upwards, while the Small line lack of response set the average offspring at the

same level as the Control. Non-linearity in this case was the consequence, rather than the cause, of the asymmetric response.

C. When considering the Control line alone, which had the most statistical power because of the large variance in parental phenotypes, the quadratic term disappeared, supporting the fact that the parent-offspring regression was linear ($h^2 \simeq 0.14, \Pr(h^2 = 0) = 0.00698, \Pr(k_2 = 0) = 0.62$)

D. Running mother-offspring and father-offspring regressions independently provided very similar results. Focusing on the control line sub-dataset, the mother-offspring regression lead to $h^2 = 0.089 \pm 0.031$ (s.e.), while the father-offspring regression resulted in $h^2 = 0.081 \pm 0.032$.
